# Supplementary material for: Comparison of Methods for Incorporating Related Data When Developing Clinical Prediction Models: A Simulation Study
Source: Biom J. 2026 Aug 27;68(5):e70166. doi: 10.1002/bimj.70166 (PMC13520826; doi:10.1002/bimj.70166)
Supplement: Supplementary file 2 [file BIMJ-68-e70166-s001.zip › third submission code review - R2/reproducibility_supplement/results/Plots/tipping_point_analysis_Noshift.pdf]

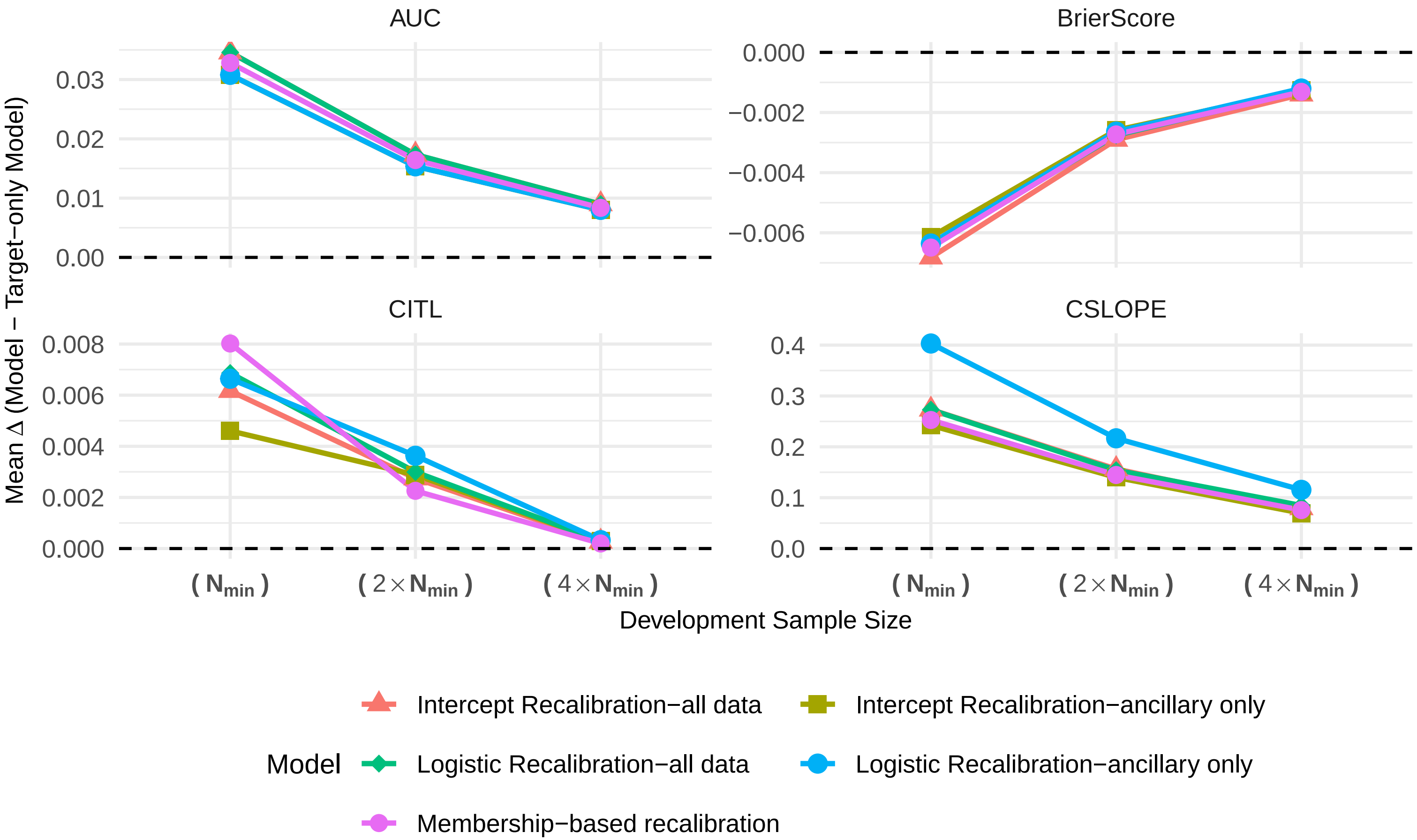

AUC
BrierScore
CITL
CSLOPE
0.03
0.02
0.01
0.00
0.000
−0.002
−0.004
−0.006
0.008
0.006
0.004
0.002
0.000
0.4
0.3
0.2
0.1
0.0
Mean Δ (Model − Target−only Model)
(N_{min})
(2×N_{min})
(4×N_{min})
Development Sample Size
Model
Intercept Recalibration−all data
Intercept Recalibration−ancillary only
Logistic Recalibration−all data
Logistic Recalibration−ancillary only
Membership−based recalibration
